# New horizons for Parkinson's disease treatment?

**DOI:** 10.1016/j.lana.2025.101103

**Published:** 2025-04-14

**Authors:** The Lancet Regional Health – Americas

Parkinson's disease (PD) remains a challenge in neurology, combining hallmark motor symptoms and a wide array of non-motor manifestations. With a multifactorial aetiology that includes genetic and environmental factors, PD is primarily characterised by bradykinesia, rigidity, tremor, and postural instability. However, non-motor symptoms such as cognitive decline, mood disorders, and autonomic dysfunction also significantly affect patients' quality of life. There is still no cure for PD, but the list of options to alleviate the symptoms is expanding fast. Current treatment options include pharmacological interventions such as levodopa and dopamine agonists, surgical options such as deep brain stimulation, and supportive therapies including physical, occupational, and speech therapy.

Last month, one of the most expected breakthroughs in PD research was published in *Science*, revealing a new horizon in the Parkinson's field and fuelling patients, family, and caregivers with hope. The study led by Dr Sylvie Callegari unveiled the structure of human ubiquitin kinase PINK1, a protein linked to the development of PD but whose structure scientists had never been able to characterise. PINK1 plays a crucial role in mitochondrial quality control, and its dysfunction leads to the accumulation of damaged mitochondria, contributing to neuronal death. Mutations in the *PINK1* gene are associated with early-onset forms of PD (EOPD), which affects individuals younger than 50 years, making this breakthrough discovery particularly important. Nevertheless, PINK1 and mitochondrial dysfunction play a role in all forms of PD, and the elucidation of its structure holds significant clinical promise, with the potential for developing disease-modifying therapies that go beyond symptomatic relief. Targeting PINK1 could potentially help preserve neuronal function and slow disease progression—the ultimate goal for neurodegenerative disease treatment.

Despite being a remarkable scientific breakthrough, the road is long and tortuous between cell biology and clinical practice, especially for PD treatments. Recent examples of promising approaches failing to show clinical efficacy for PD include two clinical trials showing the GLP-1 receptor agonist, exenatide, did not slow the rate of PD progression as in-vitro and in-vivo findings had previously suggested, and a brain-penetrant, pegylated, longer-lasting version of exenatide believed to be anti-inflammatory via reduction of microglia activation was also not better than placebo against motor or non-motor PD symptoms. Also, based on the pathophysiology of the disease, the hypothesis of low-dose ion chelators improving motor symptoms in PD was not confirmed in a multicentre trial using deferiprone in early-onset PD.

PD is the second most common neurodegenerative disease globally, with its growing prevalence mainly driven by population ageing, and the need for disease-modifying drugs and a definitive treatment will only become more urgent. A modelling study using data from the Global Burden of Disease Study estimated that 25·2 million people (95% uncertainty interval 21·7–30·1) will be living with PD by 2050, a 112% increase from 2021; and the projected age-standardised increases will be highest in east Asia and Andean Latin America. The increase in neurodegenerative diseases such as PD is incontestable; however, prevalence levels and estimates for most low-income and middle-income countries (LMICs) should be interpreted with care due to underreporting and lack of data. Using a representative cohort of the elderly population, Schlickmann and colleagues projected that the number of cases in Brazil will increase from approximately 535,000 in 2024–1,250,000 by 2060, which warrants attention. Their report also showed a high level of advanced-stage disease—a scenario likely to be reproduced in many LMICs in the region of the Americas, where primary and secondary care providers are less likely to be equipped and trained for the diagnosis.

PD diagnosis is, indeed, challenging. Since the clinicopathological component of the disease cannot be determined pre-mortem, the diagnosis is based on the presence of “typical parkinsonian motor features” in the absence of indicators suggesting alternative diagnoses. Advancing diagnosis accuracy and identification of early PD onset is a key priority for the field and essential for assisting current and future patients. Currently, PD diagnosis is resource-intensive, expensive, and dependent on highly skilled and trained personnel, explaining its inaccessibility for a large portion of the population. Scientists have tirelessly searched for biomarkers that could increase accuracy, reduce costs, and facilitate early diagnosis of PD. In the meantime, priority should be given to training all health-care providers on the clinical signs and symptoms, exposure risks, and currently available tools and guidelines for diagnosis to help improve earlier identification, especially in LMICs and minoritised populations, where late diagnosis is common. A diagnosis is not only an answer but the beginning of a journey of care that can help alleviate pain, delay disease progression, and improve quality of life, until a curative treatment is available.

Despite the lively research environment and exciting developments, PD will continue to impose a significant burden on patients, caregivers, and health-care systems for many years to come. An unavoidable issue will be to advance our health systems' preparedness and adaptation in parallel with the scientific advances to ensure we continuously provide the best care for all PD patients at all disease progression stages. Training health-care providers and developing a support system to alleviate the burden on family and caregivers, including the provision of psychological support and palliative care, are essential components of this adaptation. Emphasising preventive measures with proven links to delaying PD—such as promoting healthier diets, ensuring access to physical activity and green spaces, and enforcing regulations on pesticide use and exposure—should be prioritised and implemented. Combining our epidemiological and biological evidence will be crucial in defining clinical approaches and effective public health strategies at the local level.

As we prepare for the future, continuous support, funding, and prioritisation of translational research and clinical trials will be crucial to move knowledge from the basic to clinical to populational level and improve the lives of those living with Parkinson's and other neurodegenerative diseases.

